# Whole-Exome Sequencing and Copy Number Analysis in a Patient with Warburg Micro Syndrome

**DOI:** 10.3390/genes13122364

**Published:** 2022-12-14

**Authors:** Qiwei Wang, Tingfeng Qin, Xun Wang, Jing Li, Xiaoshan Lin, Dongni Wang, Zhuoling Lin, Xulin Zhang, Xiaoyan Li, Haotian Lin, Weirong Chen

**Affiliations:** State Key Laboratory of Ophthalmology, Zhongshan Ophthalmic Centre, Guangdong Provincial Key Laboratory of Ophthalmology and Visual Science, Guangdong Provincial Clinical Research Centre for Ocular Diseases, Sun Yat-sen University, Guangzhou 510060, China

**Keywords:** Warburg Micro syndrome, copy number variation, early diagnosis, *RAB3GAP1*

## Abstract

Warburg Micro syndrome (WARBM) is an autosomal recessive neuro-ophthalmologic syndrome characterized by microcephaly, microphthalmia, congenital cataracts, cortical dysplasia, corpus callosum hypoplasia, spasticity, and hypogonadism. WARBM is divided into four subtypes according to the causative genes, of which *RAB3GAP1* (OMIM# 602536) accounts for the highest proportion. We collected detailed medical records and performed whole-exome sequencing (WES) for a congenital cataract patient. A novel heterozygous frameshift *RAB3GAP1* variant was detected in a boy with a rare ocular phenotype of bilateral membranous cataracts accompanied by a persistent papillary membrane. Further copy number variation (CNV) analysis identified a novel deletion on chromosome 2q21.3 that removed 4 of the 24 exons of *RAB3GAP1*. The patient was diagnosed with WARBM following genetic testing. The present study expands the genotypic and phenotypic spectrum of WARBM. It suggests applying whole exome sequencing (WES) and CNV analysis for the early diagnosis of syndromic diseases in children with congenital cataracts.

## 1. Introduction

Warburg Micro syndrome (WARBM) is a rare autosomal recessive neuro-ophthalmologic syndrome characterized by microcephaly, microphthalmia, congenital cataracts (CCs), cortical dysplasia, corpus callosum hypoplasia, spasticity, and hypogonadism [1]. WARBM presents with many similar clinical disorders as Martsolf syndrome, a milder condition than WARBM. Both diseases genetically overlap and share the same molecular deficit in RAB18. According to a previous report, they were termed the Micro/Martsolf spectrum or RAB18 deficiency disorder [2]

Not only biallelic loss-of-function variants in the *RAB3GAP1* (OMIM# 602536), *RAB3GAP2* (OMIM# 609275), *RAB18* (OMIM# 602207), and *TBC1D20* (OMIM# 611663) genes reportedly cause WARBM, but it can also be caused by homozygous or compound heterozygous missense and indels variants [1,2,3,4,5]. RAB3GAP is a heterodimeric protein consisting of a 130-kDa catalytic subunit (encoded by *RAB3GAP1*) and a 150-kDa noncatalytic subunit (encoded by *RAB3GAP2*). RAB3GAP functions as a guanine–nucleotide exchange factor (GEF) for RAB18 and has a role in regulating calcium-mediated exocytosis of neurotransmitters and hormones [6]. TBC1D20 promotes RAB18 dissociation from the endoplasmic reticulum membrane into the cytosol and shows RAB18 GTPase-activating activity [7]. It is suggested that WARBM is directly caused by a loss of RAB18 or indirectly by a loss of RAB18 regulators (RAB3GAP1, RAB3GAP2, or TBC1D20) [7,8].

Here, we describe a boy with two novel *RAB3GAP1* gene variants. One of the variants is a deletion on chromosome 2q21.3, resulting in the removal of four of the twenty-four exons of *RAB3GAP1*. Our finding broadens the phenotypic and genotypic spectrum of WARBM via detailed clinical examinations and genetic screening.

## 2. Materials and Methods

### 2.1. Patients

The present study was approved by the Institutional Review Board of Zhongshan Ophthalmic Center, Sun Yat-sen University and adhered to the Helsinki Declaration. As a part of a series of ongoing studies at the Childhood Cataract Program of the Chinese Ministry of Health (CCPMOH) [9], we enrolled a patient with CCs seen in January 2021 and his parents. The proband and his parents underwent whole-exome sequencing (WES). Informed written consent was obtained from the legal guardian of the patient.

### 2.2. Clinical Assessments

Clinical data of the patient were collected based on medical history review, clinical examinations of facial dysmorphia, head circumference, height, and thorough ocular examinations including slit-lamp photography (BX900; HAAG-STREIT AG, Bern, Switzerland), Pentacam scheimpflug system (Oculus, Wetzlar, Germany), Tono-Pen (Reichert, Inc., Depew, NY, USA), and visual evoked potential (RETeval, LKC Technologies, Inc., Gaithersburg, MD, USA), and A-scan ultrasound and B-scan ultrasound (Aviso, Quantel Médical, Clermont-Ferrand, France). The horizontal corneal diameter was measured by caliper.

### 2.3. Exome Sequencing and Bioinformatic Analysis

Genomic DNA was extracted from participants’ peripheral leukocytes using a QIAamp DNA Mini Kit (Qiagen, Hilden, Germany) according to the manufacturer’s instructions. We performed WES for the proband via the Agilent v6 targeted sequence capture library process method with Illumina next-generation sequencing systems (NovaSeq 6000, Illumina Inc., San Diego, CA, USA), and bioinformatics analysis was performed as described previously [10,11]. Briefly, raw sequencing reads were matched to the human reference genome assembly (NCBI build 37/hg19) via Burrows-Wheeler Aligner. In addition, local realignment, base quality score recalibration, and variant calling (HaplotypeCaller) were performed on Toolkit (GATK). Ensembl Variant Effect Predictor with custom shell scripts (VEP, https://www.ensembl.org/vep, 6 June 2016) used to assess and annotate the variants. The frequency of variants was from the public database (1000 Genomes, dbSNP, gnomAD, ExAC, and ESP6500) and our private database. All alleles with minor allele frequencies higher than 0.01 were filtered. We then investigated the variants with detailed virtual panels by annotating the genes with PanelApp (Cataract R31, Supplementary File S1). If no candidate causative variant was detected, we further analyzed the variants from the genes included in Structural eye disease v1.3 of PanelApp (Supplementary File S2). The phenotype of the patient was further used to filter the variants. To detect copy number variations (CNVs) and sub-microscopic deletions/duplications, we used the specific software package CNVkit based on the WES data [12]. A reliable CNV reference was trained using an iterative approach, as reported previously [10]. Following the detection of a CNV, whole-genome sequencing (WGS) was performed using the BGI MGISEQ-2000 sequencer platform to confirm the existence of the CNV. Briefly, paired-end libraries were constructed using MGIEasy universal DNA library prep. The libraries were then sequenced in 2 × 150 base pair (bp) paired-end reads with at least 30× mean coverage. The Burrows-Wheeler Aligner tool (version 0.7.17) was used for alignment, and a CNVkit was applied for CNV calling. Variants were named using Human Genome Variation Society nomenclature guidelines. A total of 1000 genomes, dbSNP, ClinVar, and HGMD were checked to identify reported pathogenic variants among the candidate variants [13,14,15].

### 2.4. Pathogenicity Assessments of the Variants

The pathogenicity of the candidate variants was analyzed following the American College of Medical Genetics and Genomics/Association for Molecular Pathology (ACMG/AMP) variant classification guidelines [16,17].

## 3. Results

### 3.1. Clinical Phenotypes

**Figure 1:**
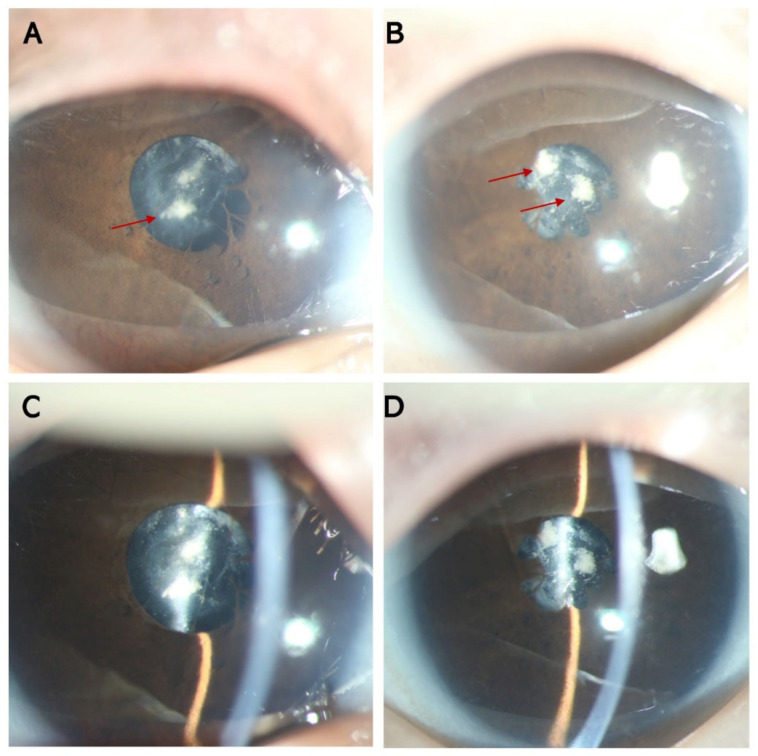
Anterior segment photographs of the patient with WARBM. (**A**) Anterior segment photograph using diffuse illumination of the right eye showing the persistent papillary membrane and lens fibrotic opacities. (**B**) Anterior segment photograph using diffuse illumination of the left eye, showing the persistent papillary membrane and lens fibrotic opacities. (**C**) Anterior segment photograph using direct focal illumination of the right eye showing the decreased thickness of the lens. (**D**) Anterior segment photograph using direct focal illumination of the left eye showing the decreased thickness of the lens. (Red arrow: lens fibrotic opacities).

The three-month-old boy presented with nystagmus, and his parents noticed whitish pupils at birth. The proband was born to non-consanguineous unaffected Chinese parents after a full-term pregnancy. No cataract-causing event occurred during pregnancy, including cytomegalovirus, herpes simplex, rubella, or toxoplasmosis infection, toxin or X-ray exposure, and relevant medication usage. No facial dysmorphia was detected. Developmental delay was also diagnosed because the boy could not lift his head at three months old. His head circumference was 35.5 cm, which indicated microcephaly, and his height was 56 cm (<97% of boys at the same age) at 3 months old [18]. Ocular examinations revealed bilateral microcornea, microphthalmos, congenital membranous cataracts, and persistent pupillary membranes. The lens was very thin, and the anterior and posterior capsules fused with fibrotic opacities (Figure 1). The corneal diameter of both eyes was 8 mm. The axial lens (AL) was 16.93 mm in the right eye and 16.68 mm in the left eye. The intraocular pressure (IOP) was 16 mmHg in the right eye and 12 mmHg in the left eye. The P2 latency of visual evoked potential was delayed in both eyes. After genetic screening, the patient was referred to a pediatric clinic for further examination and diagnosis. Magnetic resonance imaging showed corpus callosum hypoplasia, and a transcranial doppler suggested high blood pressure in the intracranial artery. The patient also had a patent foramen ovale and cryptorchidism. WARBM was then confirmed. The cataracts were removed when the boy was three months old. At the three-month follow-up, severe posterior capsular opacification was noticed in his right eye, and a second surgery was prescribed to remove the opacities. Twice yearly follow-up was scheduled.

### 3.2. Molecular Findings

**Figure 2:**
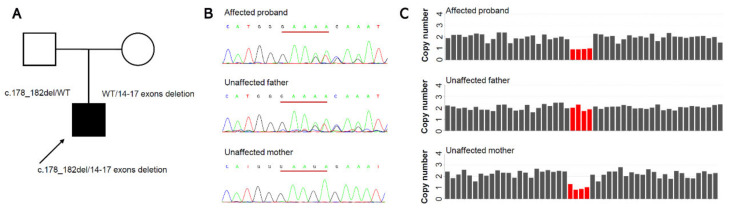
Molecular finding of the Chinese Han families. (**A**) The boy was born to non-consanguineous unaffected parents. (**B**) The sanger sequence of the family members showing a novel heterozygous c.178_182del variant in the affected proband and his unaffected father. (**C**) Chromosome 2q21.3 deletion removing 4 of the 24 exons of *RAB3GAP1* in the affected proband and his unaffected mother identified from whole exome sequencing data.

As a part of the etiology detection study at the Childhood Cataract Program of the Chinese Ministry of Health (CCPMOH), WES was performed on a patient with CCs (Figure 2A). After annotation with VEP, we performed a filter chain with Excel as follows: gnomAD_exome_ALL lower than 0.1%, gnomAD_genome_ALL lower than 1%, local variant frequency (based on almost 10,000 WES data at our sequence center) lower than 1%, gene list in OMIM morbid (20220115). Thus, we received 142 variants after the first step of filtering. Then, we further investigated the variants with detail the virtual panel by annotating the genes with PanelApp (Cataract R31). We finally obtained six variants and with only one frameshift indel of *RAB3GAP1* with VEP impact annotation HIGH. The filtered variants were also listed in Supplementary File S3.

**Figure 3:**
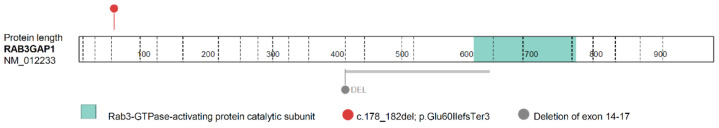
Schematic diagrams of *RAB3GAP1*. Cyan-blue: Rab3-GTPase-activating protein catalytic subunit; Red label: c.178_182del, p. Glu60IlefsTer3 variant; Grey label: chromosome 2q21.3 deletion removing 4 of the 24 exons of *RAB3GAP1*; dotted line: boundary of exons.

The c.178_182del variant (paternal origin, Figure 2B) results in the premature termination of RAB3GAP1 (p. Glu60IlefsTer3). The variant was classified as Likely Pathogenic, because it showed evidence of PVS1+PM2_support. Since *RAB3GAP1* causes autosomal recessive WARBM with the causative mechanism of loss of function (LOF), we searched for another pathogenic variant on that gene. Further CNV analysis investigated a deletion in chromosome 2q21.3 (hg19)2:135890449-135893557del (maternal origin). WGS demonstrated the existence of a deletion around chromosome (hg19)2:135888415-135895985del. The deletion removed exons 14–17 of *RAB3GAP1* (Figure 2C), with the HGVS nomenclature NM_012233.3:c.(1236+1_1237-1)_(1923+1_1924-1)del, leading to a loss of 229 amino acids (larger than 10% of the full protein length 982 amino acids). According to ACMG/AMP classification guidelines for single-gene copy number variants, we should apply PVS1_strong for this deletion. We did not find any carrier with this in-frame exon 14–17 deletion in the gnomAD SVs v2.1 population database (PM2_support), and we confirmed this exon 14–17 deletion was in-trans of the paternal c.178_182del (p.Glu60IlefsTer3) variant (PM3). According to ClinGen/ACMG/AMP Bayesian classification framework, we classed this maternal exon 14–17 deletion as likely pathogenic (PVS1_strong+PM2_support+PM3) [16,19]. The locations and domain structures (https://pecan.stjude.cloud/proteinpaint/) of the *RAB3GAP1* variants are shown in Figure 3.

## 4. Discussion

We identified a novel frameshift variant and a novel deletion of exons 14–17 in the *RAB3GAP1* gene in a proband with CCs. We described the detailed phenotype, including developmental delay, microcephaly, microphthalmia, microcornea, and congenital membranous cataracts with persistent pupillary membranes, corpus callosum hypoplasia, patent foramen ovale, and cryptorchidism. Interestingly, congenital membranous cataracts combined with persistent pupillary membranes were not mentioned in previous WARBM cases. Our findings demonstrate a severe cataract phenotype and expand the genotype spectrum of this disorder.

We identified novel compound heterozygous variants in the *RAB3GAP1* gene leading to WARBM. WARBM presents with many similar clinical disorders as Martsolf syndrome, a milder condition than WARBM. From the genotype, Martsolf syndrome is more often associated with a mutation in *RAB3GAP2*. To date, only three pathogenic variants related to Martsolf syndrome were detected in *RAB3GAP1* [20,21]. From the phenotype, Martsolf syndrome has milder clinical manifestations than WARBM. The proband showed severer clinical features including developmental delay, microcephaly, microphthalmia, microcornea, severe CCs, corpus callosum hypoplasia, patent foramen ovale, and cryptorchidism. Combining the above genotypic and phenotypic information, the patient was diagnosed with WARBM. According to previous reports [1,3,22,23,24,25], most WARBM patients originated from consanguineous families (mostly in Egypt, Pakistan, Turkey, India, Iran, and Tunisia). The disease is rare in the Chinese population because of uncommon consanguineous marriages in China. WARBM is classified into four subtypes (WARBM1–4) according to the causative gene (*RAB3GAP1*, *RAB3GAP2*, *RAB18*, and *TBC1D20*). Among them, *RAB3GAP1* accounts for the highest proportion in the population [2]. The pathological mechanisms caused by *RAB3GAP1* variants are only partially understood. RAB3GAP1 is the catalytic subunit of the RAB3GAP heterodimeric complex, which acts as a GEF to activate RAB18. To date, more than 65 variants of the *RAB3GAP1* gene have been reported, including splice site, nonsense, frameshift mutations, and micro deletion. Most of the above variants were predicted to cause nonsense-mediated mRNA decay and protein LOF with autosomal recessive inheritance [3]. The LOF of the RAB3GAP complex indirectly caused RAB18 deficiency via the loss of its regulators. RAB18 is ubiquitously expressed, has particularly high expression in the brain and epithelia, and regulates the exocytosis of neurotransmitters and hormones [6]. A RAB18 deficiency might impair specific vesicle transport routes, causing WARBM. We suggest the paternal origin c.178_182del variant in the present study caused the disease through the above mechanism. For the in-frame deletion of *RAB3GAP1* exons 14–17, it leads to producing a shorter protein, with a loss of 229 amino acids, than wildtype RAB3GAP1, affecting the integrity of the Rab3-GTPase-activating protein catalytic subunit. The function of the mutated RAB3GAP1 as the GEF for RAB18 might be impaired. Therefore, WARBM occurred. A congenital membranous cataract is an especially severe type of CC, impairing lens transparency and morphology. The lens material was absorbed, leading to a decrease in lens thickness. The anterior and posterior capsules gradually fused and became cloudy. The severe disorders described above suggest that *RAB3GAP1* gene mutations cause significant damage to lens development.

Cataracts can be directly observed through the transparent cornea and pupil, allowing early detection. Additionally, the lens is sensitive to whole-body homeostasis disorders because of its selectively permeable lens capsule, the nuclear degradation of lens fibers, and slow lens protein turnover [26]. Therefore, cataracts could be the first manifestations of many syndromes. Since infants are challenging to examine and many manifestations of WARBM are insidious and uncharacteristic, they are often initially misdiagnosed as simple cataracts. However, genetic testing allows children with syndromic diseases to be diagnosed promptly, and their prognosis might be improved with early rehabilitation. For our patient, genetic screening not only helps the present patient have an earlier diagnosis and treatment but also makes genetic counseling and prenatal diagnosis possible.

WES is a convenient and fast method of initial genetic screening for CCs. Most inherited cataracts are caused by small-scale mutations, including substitutions, insertions, and deletions [27]. WES is reliable and efficient in detecting these variants, with a high diagnostic yield up to 85% [28,29,30,31,32], and is therefore used for the initial genetic screening for CCs. Among the patients without causative variants identified during the initial genetic screen, we searched for larger-scale mutations using CNV analysis based on the WES data. Previously, array comparative genomic hybridization and multiplex ligation-dependent probe amplification proved CNV detection means with high accuracy [33]. However, they are not suggested as the first-tier diagnostic test for CCs because small-scale mutations represent a significant proportion of pathogenic variants of CCs. In contrast, WES is particularly designed to identify those variants. Additionally, CNV detection by WES has become increasingly precise due to capture and library progression, coverage and uniformity improvement, and bioinformatics software development. WES can accurately identify CNVs larger than 1 Mb with high specificity and sensitivity [34]. Therefore, we recommend using WES as the first-tier diagnostic test for CCs and including CNV analysis to improve the overall diagnostic rate. In the present case, we performed additional WGS to verify CNVs and explore the deletion breakpoint. The maternal deletion of *RAB3GAP1* exons 14–17 could be detected with a CNVkit and read depth signatures by trio WGS. However, the exact breakpoints of the maternal CNV were not detected with split-reads. Using the UCSC genome browser [35], we found tandem repeat and low mappability regions spread across multiple introns of the *RAB3GAP1* gene, leading to uncertainty about the precise CNV breakpoint of the short reads sequencing technology.

Limitations also exist in our study, there was no experimental GEF assay for the identified variants since RAB3GAP functions as a GEF for RAB18. A methodologically sound study is needed to further confirm the mechanism.

## 5. Conclusions

This study expands the genotypic and phenotypic spectrum of WARBM. We identified novel compound heterozygous variants (c.178_182del, p. Glu60IlefsTer3 and deletion of exons 14–17) in *RAB3GAP1* based on WES data. Genetic screening played a significant role in the early diagnosis of syndromic diseases in children with CCs.

## Data Availability

The data are available from the corresponding authors upon reasonable request.

## Supplementary Materials

The following supporting information can be downloaded at: https://www.mdpi.com/article/10.3390/genes13122364/s1, Supplementary File S1: PanelApp (Cataract R31); Supplementary File S2: PanelApp (Structural eye disease v1.3); Supplementary File S3: The list of filtered variants.
